# Ca^2+^ ionophores are not suitable for inducing mPTP opening in murine isolated adult cardiac myocytes

**DOI:** 10.1038/s41598-017-04618-4

**Published:** 2017-06-27

**Authors:** Mathieu Panel, Bijan Ghaleh, Didier Morin

**Affiliations:** 1 INSERM U955, équipe 03, Créteil, France; 2 Université Paris-Est, UMR S955, DHU A-TVB, UPEC, Créteil, France

## Abstract

Opening of the mitochondrial permeability transition pore (mPTP) plays a major role in cell death during cardiac ischaemia-reperfusion. Adult isolated rodent cardiomyocytes are valuable cells to study the effect of drugs targeting mPTP. This study investigated whether the use of Ca^2+^ ionophores (A23187, ionomycin and ETH129) represent a reliable model to study inhibition of mPTP opening in cardiomyocytes. We monitored mPTP opening using the calcein/cobalt fluorescence technique in adult rat and wild type or cyclophilin D (CypD) knock-out mice cardiomyocytes. Cells were either treated with Ca^2+^ ionophores or subjected to hypoxia followed by reoxygenation. The ionophores induced mPTP-dependent swelling in isolated mitochondria. A23187, but not ionomycin, induced a decrease in calcein fluorescence. This loss could not be inhibited by CypD deletion and was explained by a direct interaction between A23187 and cobalt. ETH129 caused calcein loss, mitochondrial depolarization and cell death but CypD deletion did not alleviate these effects. In the hypoxia-reoxygenation model, CypD deletion delayed both mPTP opening and cell death occurring at the time of reoxygenation. Thus, Ca^2+^ ionophores are not suitable to induce CypD-dependent mPTP opening in adult murine cardiomyocytes. Hypoxia-reoxygenation conditions appear therefore as the most reliable model to investigate mPTP opening in these cells.

## Introduction

Necrosis is a prevalent form of cell death that contributes to cell injuries in a number of diseases. This is particularly relevant during cardiac ischaemia-reperfusion^1, 2^ where opening of the mitochondrial permeability transition pore (mPTP) has been shown to play a major role^3^. mPTP is thought to be a multiprotein complex which forms and opens under conditions that prevail at the time of reperfusion such as Ca^2+^ overload and oxidative stress^4^. Its opening causes mitochondrial swelling, loss of membrane potential, ATP depletion and outer membrane permeabilization leading to cell death. The precise components of the pore remain putative. Recent data provided evidence in support of a role for the F_1_F_0_ ATP synthase in pore formation^5, 6^ although there is no agreement in the mechanism of pore formation. Nevertheless, a common agreement considers that the soluble protein cyclophilin D (CypD) located within the mitochondrial matrix is the main regulator of mPTP as CypD lowers the Ca^2+^ threshold required to elicit mPTP opening. Indeed, excess intramitochondrial Ca^2+^ is a crucial factor to trigger mPTP opening^7^. This is the reason why numerous studies have used Ca^2+^ ionophores to induce mPTP opening in a variety of cells^8–11^. This is a useful approach to study the effect of drugs interacting with mPTP and the search for new mPTP inhibitors remains a major pharmacological objective.

The aim of this study was to investigate whether three Ca^2+^ ionophores, A23187, ionomycin and ETH129 might represent useful tools to study mPTP opening/inhibition in excitable cells such as cardiomyocytes where Ca^2+^ fluxes have a critical role. On one hand, A23187 and ionomycin are carboxylic acid ionophores which equilibrate Ca^2+^ concentrations across biological membranes and thus between the different compartments of the cell in an electroneutral manner^12^. On the other hand, ETH129 is a neutral compound which binds Ca^2+^ with a high specificity over other divalent cations. The complex formed carries a positive charge and permeates membranes in response to negative membrane potential^13, 14^. We used the calcein loading CoCl_2_ quenching technique^15^ to monitor mPTP opening and compared the effect observed in adult cardiomyocytes isolated from rat as well as wild type and CypD knock-out mice. We observed that among the three ionophores tested, ionomycin was without effect on calcein or TMRM fluorescence and that A23187 and ETH129 modified the distribution of calcein in cardiomyocytes independently from CypD and thereby are not valid tools to monitor mPTP in isolated cardiomyocytes. In contrast, hypoxia-reoxygenation was responsible for a CypD-dependent mPTP opening as demonstrated by the loss of mitochondrial calcein and the subsequent cell death. This setting appeared as the most suitable model to investigate mPTP opening in cardiomyocytes.

## Results

### Electroneutral Ca^2+^ ionophores and mPTP opening in isolated adult cardiomyocytes

#### Induction of mPTP opening by A23187 and ionomycin in isolated rat cardiac mitochondria

**Figure 1:**
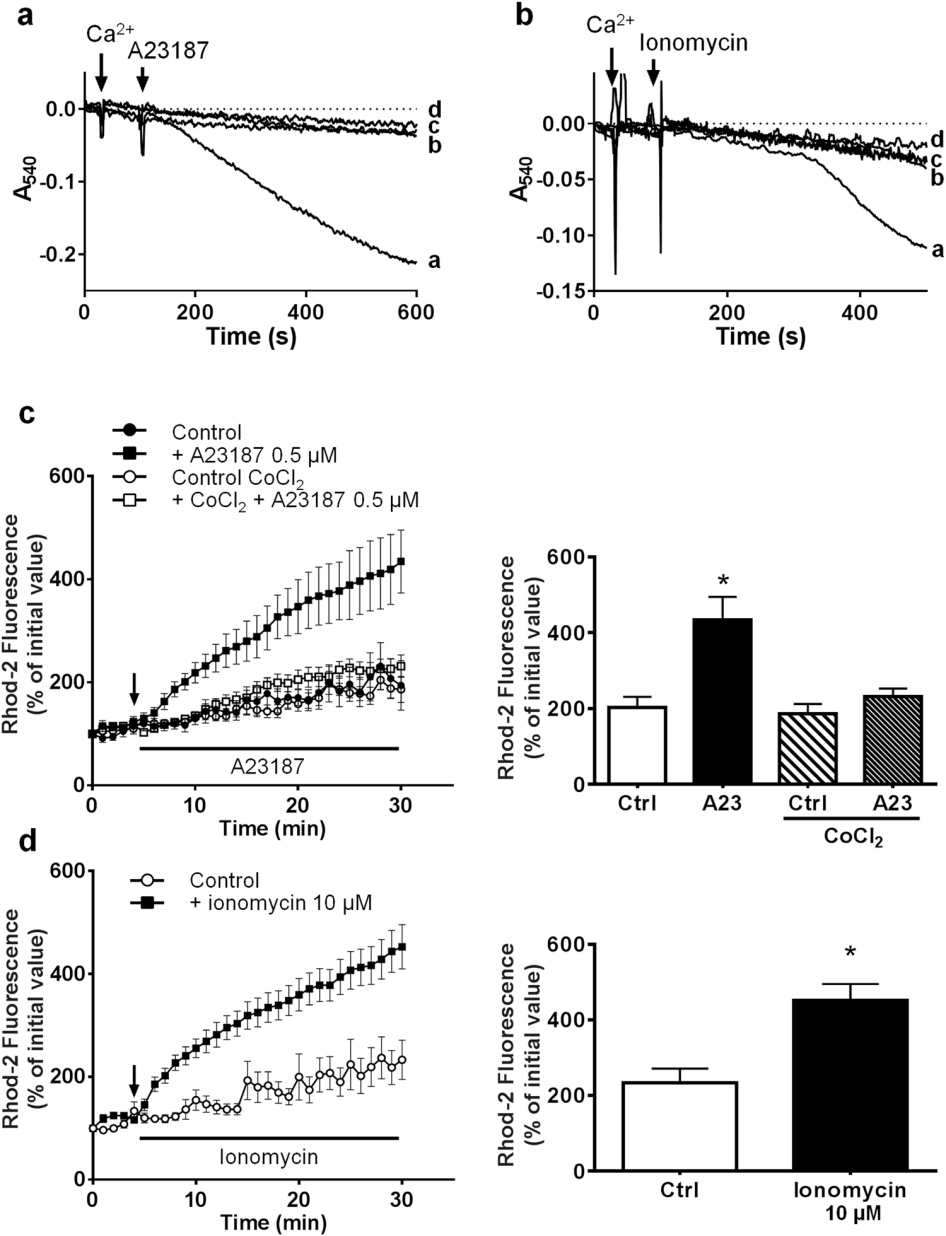
Effect of A23187 and ionomycin on mitochondrial swelling and mitochondrial Ca^2+^ in isolated cardiomyocytes. (**a** and **b**) Swelling was assessed in isolated rat cardiac mitochondria by measuring the change in absorbance of the mitochondrial suspension at 540 nm. Mitochondria were incubated in the presence of rotenone (1 µM) and antimycin A (1 µM) in order to inhibit mitochondrial respiration. Then 200 µM Ca^2+^ and 1 µM A23187 or 10 µM ionomycin were added. (**a**) line a: Ca^2+^ + A23187; line b: Ca^2+^ + A23187 + CsA 2 µM; line c: Ca^2+^ alone; line d: A23187 alone. (**b**) line a: Ca^2+^ + ionomycin; line b: Ca^2+^  + ionomycin + CsA 2 µM; line c: Ca^2+^ alone; line d: ionomycin alone. (**c**,**d**) Isolated adult cardiomyocytes were loaded with 5 µM rhod-2 for 40 min at 37 °C and then incubated in a Tyrode’s buffer. After 5 min incubation (arrow) A23187 (**c**) or ionomycin (**d**) were added to the medium. Fluorescence values were obtained by averaging pixel intensities after background substraction and were normalised as a percentage of the initial value. Each trace represents the mean ± SEM of 3–5 experiments. *p < 0.05 versus control (Ctrl). The ordinates of the bar graphs are the values of rhod-2 fluorescence observed at 30 min in the corresponding figures.

Cardiac mitochondria were incubated in the presence of rotenone (1 µM) and antimycin A (1 µM) in order to inhibit mitochondrial respiration. In these conditions, the lack of mitochondrial membrane potential limited Ca^2+^ influx and no swelling could be observed after addition of a high Ca^2+^ concentration (200 µM). A23187 (1 µM) and ionomycin (10 µM) allowed Ca^2+^ to equilibrate across mitochondrial membranes and triggered a slow mitochondrial swelling (Fig. 1a and b). This swelling could be inhibited by 2 µM CsA, a well-known de-sensitiser of mPTP opening, demonstrating that both drugs induced a CypD-dependent mPTP opening in isolated cardiac mitochondria.

#### Mitochondrial Ca^2+^ mobilization by A23187 and ionomycin in isolated adult cardiomyocytes

Then, we verified the ability of both ionophores to induce mitochondrial Ca^2+^ influx in cells. Mitochondrial compartment of isolated adult cardiomyocytes were preloaded with Rhod-2 at 37 °C for 40 min, and then supplemented with 0.5 µM A23187 or 10 µM ionomycin. Figure 1c and d show that both drugs induced a time-dependent increase in mitochondrial Ca^2+^ confirming the ionophoretic properties of the drugs.

#### A23187 but not ionomycin induced mitochondrial calcein loss

**Figure 2:**
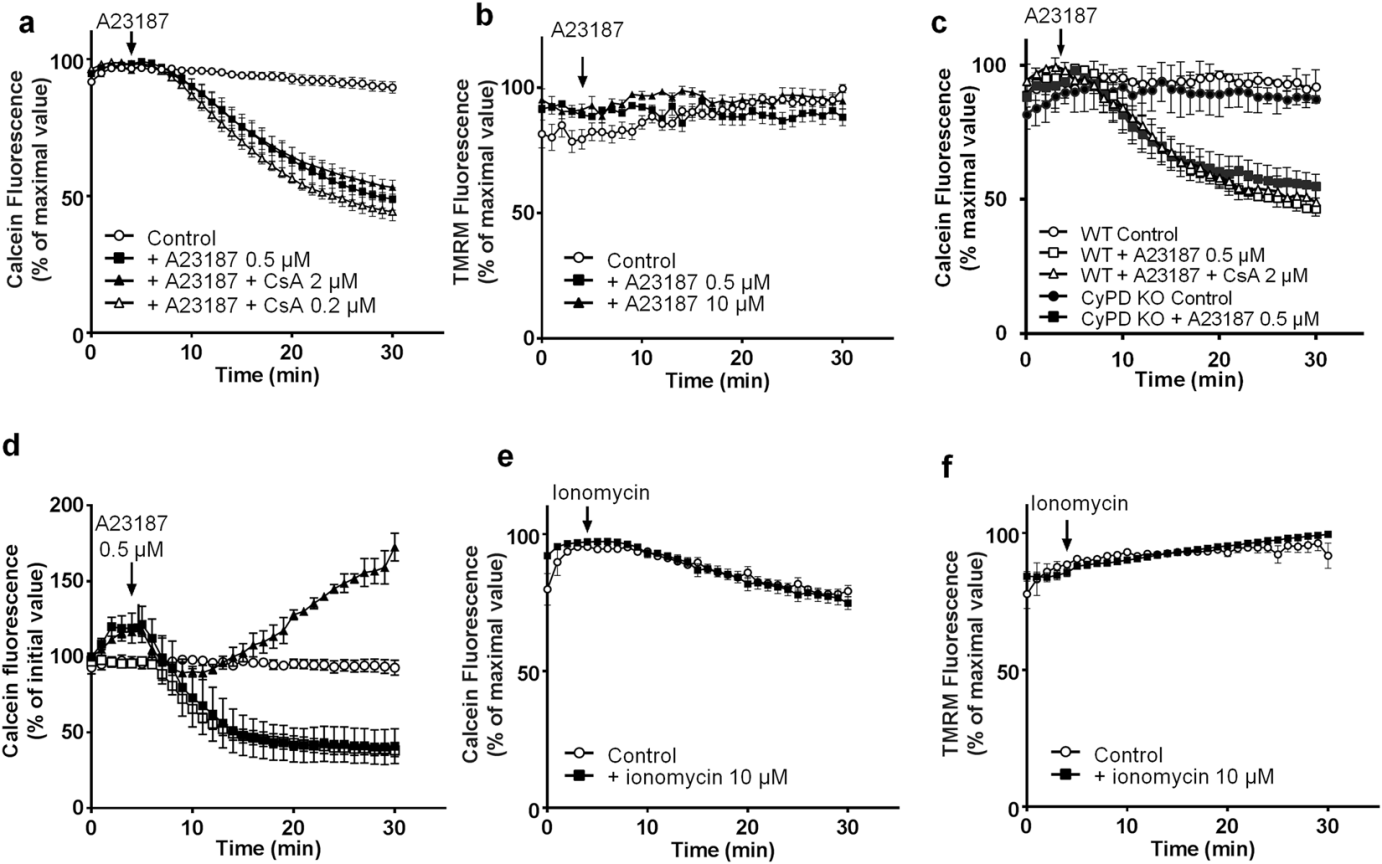
Effect of A23187 and ionomycin on mitochondrial calcein and TMRM fluorescence in isolated cardiomyocytes. Isolated adult cardiomyocytes were co-loaded with calcein, CoCl_2_ and TMRM at 37 °C and then incubated in a Tyrode’s buffer. After 5 min incubation A23187 (**a** and **b**) or ionomycin (**e** and **f**) were added to the medium. (**c**) Cardiomyocytes isolated from wild type (WT) or CypD knock out (KO) mice were stained with 1 µM calcein in the presence of 1 mM CoCl_2_, pre-treated with 2 µM CsA and 0.5 µM A23187 was added 5 min after recording. (**d**) Cardiomyocytes were incubated in a Tyrode’s buffer without Ca^2+^ (▪), supplemented with 100 µM EGTA (⚪, ▴) or containing 1.3 mM Ca^2+^ (◽). At 5 min, 0.5 µM A23187 was added (except⚪). Fluorescence values of calcein and TMRM were obtained by averaging pixel intensities after background substraction and were normalised as a percentage of the initial value. Each trace represents the mean ± SEM of 3 experiments.

We next investigated the ability of the Ca^2+^ ionophores to induce mPTP opening in isolated rat adult cardiomyocytes. The cells were co-loaded with 1 µM calcein in the presence of CoCl_2_ and TMRM and incubated at 37 °C. After 5 min incubation, the addition of 0.5 µM A23187 resulted in an immediate decrease in calcein fluorescence (Fig. 2a). This decrease occurred simultaneously in all cells and was not associated with mitochondrial depolarization as assessed by the stability of the TMRM signal (Fig. 2b). No signs of cell death were observed up to 60 min of incubation (not shown). Similar results were obtained with a higher concentration of A23187 (10 µM). When cells were incubated with CsA (0.2–2 µM) for 20 min prior to A23187 exposure, calcein fluorescence loss was not modified as compared to control cells (Fig. 2a). The same response to A23187 was obtained in cardiomyocytes isolated from wild type (WT) mice, with an immediate drop in calcein fluorescence which could not be limited by 2 µM CsA (Fig. 2c). These results indicate that mPTP is not involved in the loss of calcein fluorescence. However, since CsA has a narrow range of action, being ineffective at low and toxic at high concentrations^16–18^, we performed the same experiments on cardiac myocytes isolated from CypD knock-out mice which are protected from mPTP opening^19^. In these cells A23187 induced calcein fluorescence loss in a same extent than in WT mice, confirming a mechanism independent from CypD and unrelated to mPTP opening (Fig. 2c).

To study the mechanism of calcein release from mitochondria, we performed experiments in a Tyrode’s medium devoid of Ca^2+^. The absence of Ca^2+^ in the incubation medium did not modify the decrease in calcein fluorescence induced by A23187 (Fig. 2d). Moreover, when EGTA (100 µM) was added to the medium, A23187 caused an increase in calcein fluorescence (Fig. 2d). As EGTA does not enter the cell and is able to bind cobalt^20^, these data suggest that the increase in calcein fluorescence could be due to a possible cobalt efflux (de-quenching). Hence, we hypothesized that the decrease in calcein fluorescence was the consequence of an interaction between A23187 and cobalt instead of Ca^2+^. The experiment of Fig. 1c confirmed this hypothesis. Indeed, mitochondrial Ca^2+^ influx was inhibited when cardiomyocytes were pre-treated with 1 mM CoCl_2_. Therefore in our experimental conditions (Tyrode’s medium containing 1.3 mM Ca^2+^) A23187 might transport cobalt across mitochondrial membranes inducing the quenching of calcein fluorescence inside mitochondria without triggering mPTP opening.

The ineffectiveness of electroneutral Ca^2+^ ionophores to induce mPTP opening in cardiomyocytes was confirmed using ionomycin. Although like A23187, high concentrations of ionomycin have the capacity to elicit a CypD-dependent mitochondrial swelling in isolated cardiac mitochondria (Fig. 1b) and to produce mitochondrial Ca^2+^ overload in cardiomyocytes (Fig. 1d), ionomycin altered neither calcein nor TMRM mitochondrial fluorescence (Fig. 2e and f).

### ETH129 and mPTP opening in isolated adult cardiomyocytes

#### ETH129 induced calcein and TMRM loss and cell death independently from mPTP opening in isolated adult cardiomyocytes

**Figure 3:**
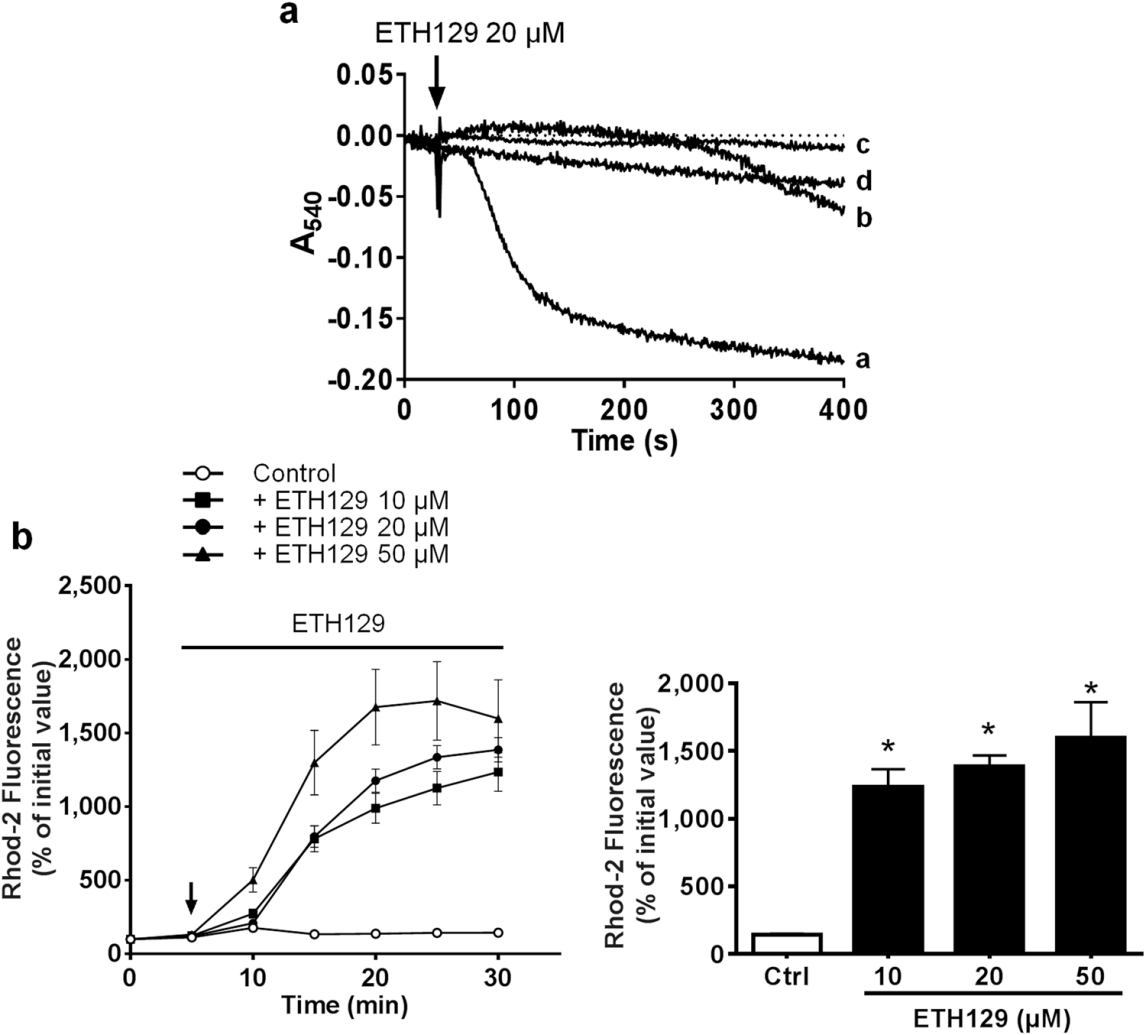
Effect of ETH129 on mitochondrial swelling and mitochondrial Ca^2+^ in isolated cardiomyocytes. (**a**) Swelling was assessed in isolated rat cardiac mitochondria by measuring the change in absorbance of the mitochondrial suspension at 540 nm. Mitochondria were energised with pyruvate/malate (5/5 mM) in the presence of 10 µM ruthenium red and 200 µM Ca^2+^. Then 20 µM ETH129 were added (arrow). Line a: Ca^2+^ + ETH129; line b: Ca^2+^  + ETH129 + CsA 2 µM; line c: Ca^2+^ alone; line d: ETH129 alone. (**b**) Isolated adult cardiomyocytes were loaded with 5 µM rhod-2 for 40 min at 37 °C and then incubated in a Tyrode’s buffer. After 5 min incubation (arrow) ETH129 was added to the medium. Fluorescence values were obtained by averaging pixel intensities after background substraction and were normalised as a percentage of the initial value. Each trace represents the mean ± SEM of 3–5 experiments. *p < 0.05 versus control (Ctrl). The ordinates of the bar graphs are the values of rhod-2 fluorescence observed at 30 min.

The Ca^2+^ ionophore ETH129 was shown to induce mPTP opening in yeast^21^ and therefore we investigated its effect on isolated murine cardiomyocytes. First, we determined whether ETH129 was able to induce mitochondrial swelling in isolated mitochondria. Its mechanism of action differs from that of A23187 as it involves an electrophoretic transport of Ca^2+^ and requires a negative potential to exert its ionophore activity. Thus, swelling experiments were performed on energised mitochondria in which the mitochondrial Ca^2+^ uniporter was blocked by the presence of ruthenium red. Figure 3a shows that 20 µM ETH129 induced mitochondrial swelling which was inhibited by CsA, indicating that ETH129 induced mPTP opening in isolated mitochondria.

Then, we checked the ability of ETH129 to trigger mitochondrial Ca^2+^ influx in cardiomyocytes. As shown in Fig. 3b, the addition of increasing concentrations of ETH129 to cardiomyocytes induced a mitochondrial uptake of Ca^2+^ as demonstrated by the increase in rhod-2 fluorescence.

**Figure 4:**
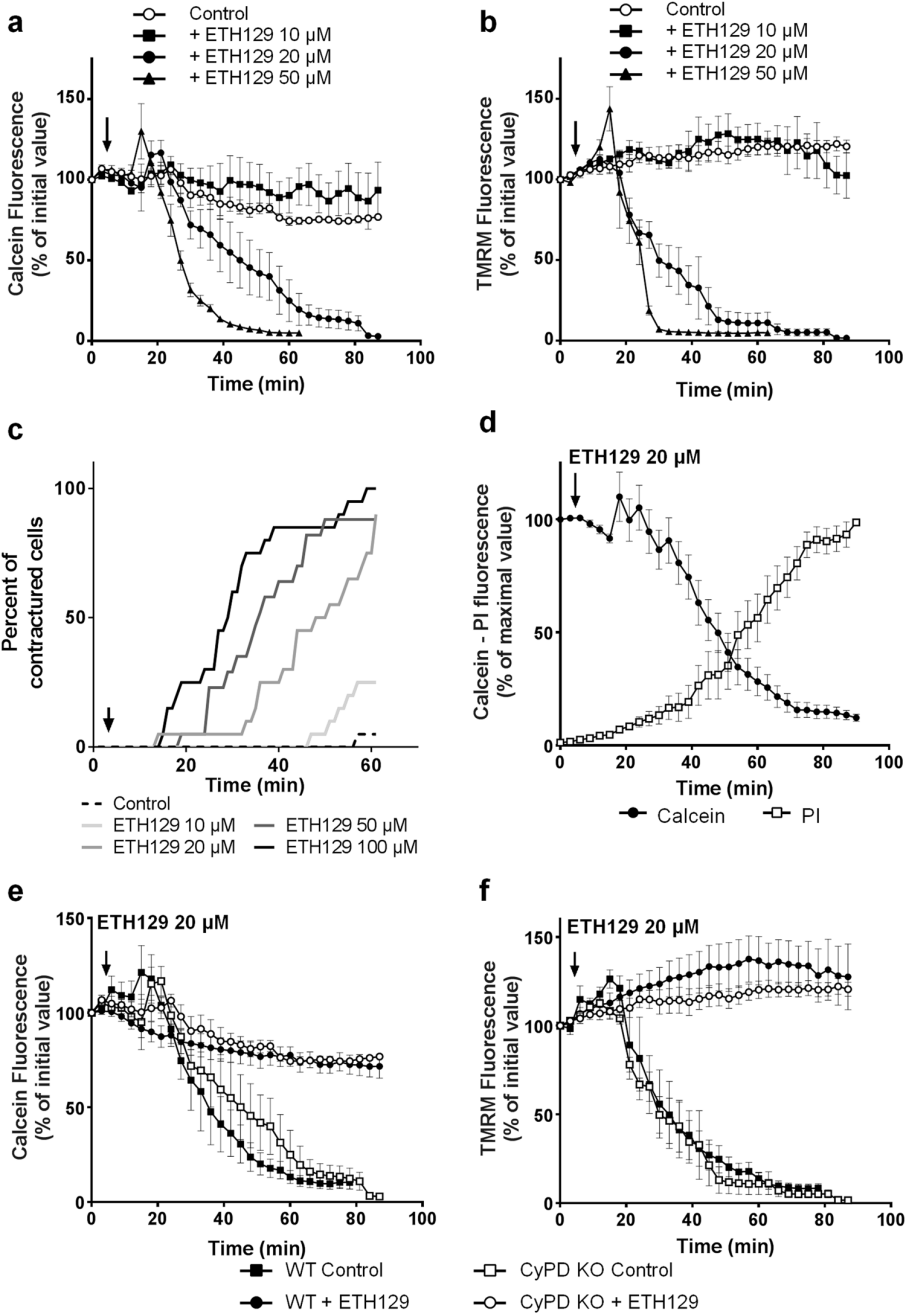
Effects of ETH129 on isolated mouse cardiomyocytes. (**a** and **b**) Isolated mouse adult cardiomyocytes were co-loaded with calcein, CoCl_2_ and TMRM at 37 °C and then incubated in a Tyrode’s buffer. After 5 min incubation ETH129 (arrow) was added to the medium. (**c**) ETH129 induces a concentration-dependent hypercontracture of cardiomyocytes. (**d**) Isolated mouse adult cardiomyocytes were co-loaded with calcein, CoCl_2_ and propidium iodide (PI) at 37 °C and then incubated in a Tyrode’s buffer. After 5 min incubation ETH129 (20 µM; arrow) was added to the medium. (**e** and **f**) Cardiomyocytes isolated from wild type (WT) or CypD knock out (KO) mice were co-loaded with calcein, CoCl_2_ and TMRM at 37 °C and then incubated in Tyrode’s buffer. After 5 min incubation, 20 µM ETH129 (arrow) was added to the medium. Fluorescence values were obtained by averaging pixel intensities after background substraction and were normalised as a percentage of the initial value. Each trace represents the mean ± SEM of 3–5 experiments.

We next tested whether ETH129 was able to induce mPTP opening in isolated cardiomyocytes. Figure 4a shows that ETH129 induced a time-dependent release of calcein which was observed at 20 and 50 µM ETH129. As opposed to A23187, calcein release is preceded by mitochondrial depolarization shown by the loss of TMRM (Fig. 4b) that followed a concentration-dependent hypercontracture of the cells (Fig. 4c). Co-loading of the cells with calcein and propidium iodide demonstrated that the loss of calcein induced by 20 µM ETH129 (T_mPTP50_ = 49 ± 15 min) led to cell death (T_cell death50_ = 56 ± 16 min) (Fig. 4d). All these data converged to indicate that ETH129 induced mPTP opening.

To confirm this effect, the same experiments were duplicated in cardiomyocytes isolated from CypD knock-out mice. In these cells the absence of CypD was assumed to inhibit mPTP opening. Figure 4e and f show that the decrease in calcein and TMRM fluorescence induced by 20 µM ETH129 in CypD knock out cardiomyocytes was similar to that observed in cardiomyocytes isolated from wild type mice, *i.e*., suggesting a CypD-independent mPTP opening process.

**Figure 5:**
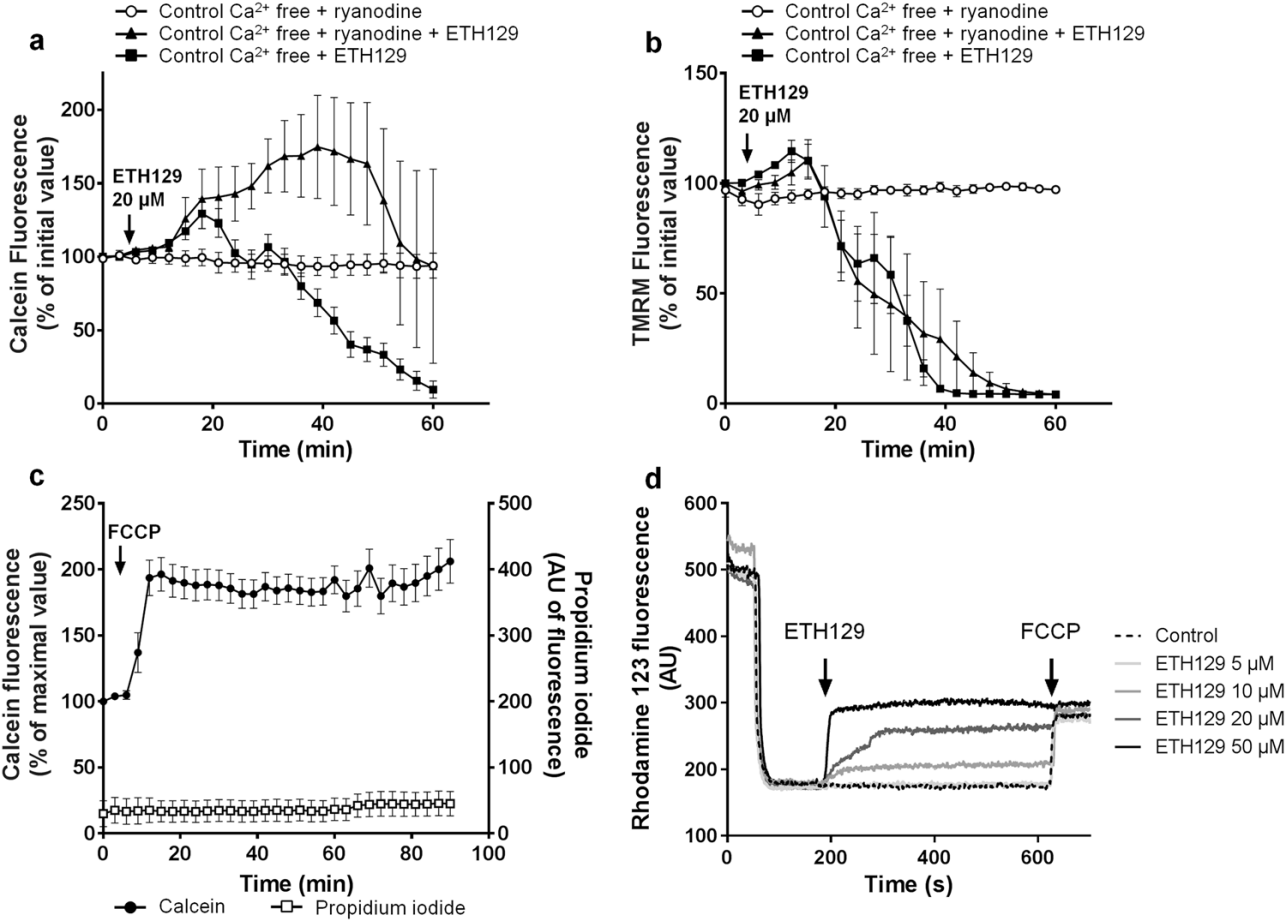
The role of Ca^2+^ and of uncoupling in the effect of ETH129. (**a** and **b**) Isolated mouse adult cardiomyocytes were co-loaded with calcein, CoCl_2_ and TMRM at 37 °C and then incubated in a Tyrode’s buffer. After 5 min incubation, 20 µM ETH129 (arrow) was added to the medium. Prior to experiments, cells were incubated for 40 min in Tyrode’s medium devoid of Ca^2+^ supplemented with 20 nM ryanodine to deplete sarcoplasmic reticulum Ca^2+^ stocks. (**c**) Isolated mouse adult cardiomyocytes were co-loaded with calcein, CoCl_2_ and propidium iodide at 37 °C and then incubated in a Tyrode’s buffer. After 5 min incubation 1 µM FCCP (arrow) was added to the medium. Fluorescence values were obtained by averaging pixel intensities after background substraction and were normalised as a percentage of the initial value. Each trace represents the mean ± SEM of 3–5 experiments. (**d**) Mitochondrial membrane potential in isolated cardiac mitochondria treated with increasing concentrations of ETH129 was followed by measuring the uptake/release of the fluorescent dye rhodamine 123. Control: 1 µM FCCP was added to depolarise mitochondrial membrane potential. Curves are representative of at least 4 independent experiments.

To further decipher the mechanism of action of ETH129, we analyzed the role of Ca^2+^. The absence of Ca^2+^ in the incubation medium did not modify the effect of 20 µM ETH129 on calcein and TMRM fluorescence (Fig. 5a and b) indicating that extracellular Ca^2+^ was not involved. Then cardiomyocytes were incubated for 40 min in a Tyrode’s medium devoid of Ca^2+^ supplemented with low concentration of ryanodine (20 nM) in order to deplete sarcoplasmic reticulum Ca^2+^ stocks^22^. In these conditions, the effect of ETH129 was associated with a delay in calcein loss as compared to control cells (Fig. 5a) but intracellular Ca^2+^ depletion did not affect mitochondrial depolarization (Fig. 5b) and hypercontracture of the cells (not shown). This suggests that the mobilization of intracellular Ca^2+^ promoted the loss of calcein caused by 20 µM ETH129 but that another mechanism was responsible for mitochondrial depolarization and cellular hypercontracture.

#### ETH129 displays mitochondrial uncoupling properties

It is well-established that mitochondrial depolarization and cellular hypercontracture are associated with ATP depletion and can be promoted by mitochondrial uncoupling^23^. We observed the same behaviour in our experimental conditions since the uncoupling agent FCCP elicited hypercontracture of the cells resulting in an increase in calcein fluorescence (Fig. 5c). Therefore, we assessed whether the mechanism of ETH129 could be linked to changes in mitochondrial potential.

For this purpose, isolated rat cardiac mitochondria were energised with pyruvate/malate and membrane potential was assessed with rhodamine 123. At low concentrations (≤10 µM), ETH129 did not modify mitochondrial membrane potential. Treatment of energised mitochondria with increasing concentrations of ETH129 (10–50 µM) resulted in a concentration-dependent depolarization and at 50 µM, ETH129 immediately collapsed membrane potential as effectively as FCCP (Fig. 5d). Thus, ETH129 has a direct uncoupling effect which explains mitochondrial depolarization and cell hypercontracture but not calcein loss or cell death since they were not altered by depolarization (Fig. 5c).

### Hypoxia-reoxygenation: a relevant model to monitor mPTP opening in isolated cardiomyocytes

**Figure 6:**
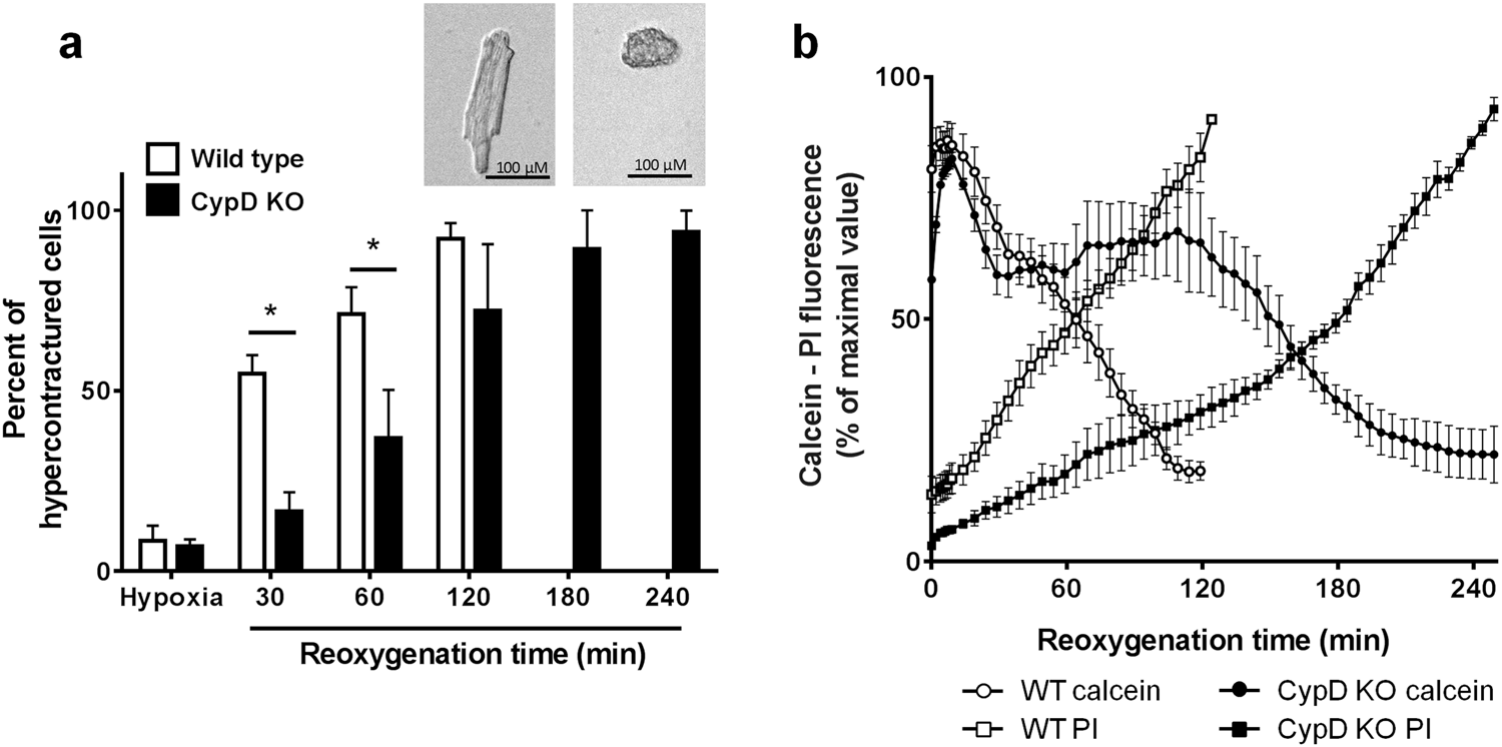
Effect of CypD deletion on hypercontracture, mPTP opening and cell death after 45 min hypoxia followed by reoxygenation in isolated adult mouse cardiomyocytes. Cardiomyocytes were isolated from wild type (WT) and CypD knock-out (KO) mice, were co-loaded with calcein, CoCl_2_ and propidium iodide (PI) and were subjected to 45 min hypoxia and then reoxygenated. Images were collected every min during the first 5 min of reoxygenation and then every 5 min. (**a**) Percentage of cells exhibiting hypercontracture at the end of hypoxia and at different reoxygenation times. (**b**) Time course of mPTP opening and cell death during reoxygenation. Fluorescence was normalised to 100% of the maximal values. Traces from 3 to 5 experiments were averaged.

The conditions required to induce mPTP opening are those encountered by a mitochondria when an ischemic organ is subjected to reperfusion and this can be mimicked in cells using a sequence of hypoxia-reoxygenation. Figure 6 shows the results obtained with mouse cardiomyocytes challenged with 45 min hypoxia followed by reoxygenation. During the hypoxic period, only a small percentage (~10%) of cells exhibited hypercontracture and no cell death was observed. Hypercontracture was unrelated to CypD as there is no difference between wild type and CypD knock out mice (Fig. 6a). Most of the damages were observed during reoxygenation. Indeed, Fig. 6a shows the time dependent occurrence of hypercontracture which was delayed in CypD knock out mice. In addition, in wild type mice, reoxygenation induced a loss of calcein (t_1/2 = _63 ± 17 min) that preceded propidium iodide labelling of cells by a few minutes (Fig. 6b). The deletion of CypD greatly delayed calcein loss (t_1/2_ = 144 ± 34 min) and propidium iodide labelling. For instance, after 120 min reperfusion, whereas 80% of cardiomyocytes were still alive in the CypD deleted cells, nearly all the cells were dead in wild type cells. This demonstrates that these experimental conditions allow to monitor a CypD-dependent mPTP opening, leading to cell death in mouse cardiomyocytes.

## Discussion

Numerous experimental protocols have been described to monitor mPTP opening in mitochondria isolated from different organs but the demonstration of its occurrence in cells is more difficult. The development of the calcein loading CoCl_2_ quenching technique based on the monitoring of the mitochondrial fluorescence of calcein allowed the investigation of mPTP opening at the single cell level^15, 24^. This technique has been used to evaluate mPTP opening in different cells and pathological situations^25–29^ but also to investigate the regulation of the pore. Different biophysical methods or pharmacological agents modulating oxidative stress or Ca^2+^ fluxes have been used to induce mPTP opening^25, 30, 31^.

In the present study, we have evaluated the use of Ca^2+^ ionophores to induce mPTP opening in cardiomyocytes which would represent a simple tool to identify potential mPTP inhibitors. We used freshly isolated murine adult cardiomyocytes because they are fully differentiated and can be electrically paced, *i.e*., a more physiological setting than cultured cells. We investigated the effects of two families of Ca^2+^ ionophores. The first one includes A23187 and ionomycin which belong to the carboxylic acid ionophores. Such compounds allow the equilibration of Ca^2+^ concentrations across biological membranes by exchanging one Ca^2+^ for 2 H^+^ in an electroneutral manner. The second one, ETH129, is a neutral compound which binds Ca^2+^ with a high specificity over other divalent cations. The complexed form of ETH129, which carries a positive charge, permeates membranes in response to negative potential. Several studies demonstrated that ETH129 transports Ca^2+^ across biological membranes^13, 14^ and induces mPTP opening in mitochondria isolated from *Saccharomyces cerevisiae* ^22^ but to our knowledge, ETH129 has never been used in mammalian cells.

Our data demonstrate that A23187 and ionomycin were unable to trigger mPTP opening in cardiomyocytes. They did not alter mitochondrial membrane potential or cell shape. The immediate and large calcein loss from cardiomyocytes challenged with A23187 was unrelated to mPTP opening. Indeed, this calcein loss could not be inhibited either by CsA treatment or CypD deletion. These results also rule out the possibility that calcein loss could be due to transient mPTP opening^32, 33^. Our experiments suggest that A23187 is able to transport cobalt across membranes and therefore the decrease in calcein fluorescence is due to mitochondrial cobalt entry and not to mPTP opening in our experimental conditions. However a number of studies have used these drugs to trigger mPTP^8–11, 31, 34, 35^ and in the present study, isolated de-energised cardiac mitochondria treated with A23187 or ionomycin underwent a CsA-sensitive swelling. A possible explanation for this discrepancy is that the mitochondrial matrix Ca^2+^ concentration reached in the presence of these drugs is not sufficient to induce mPTP opening in cardiac mitochondria. Indeed, experiments performed with isolated cardiac mitochondria demonstrate that they are very resistant to Ca^2+^ overload and able to take up huge Ca^2+^ concentrations before mPTP opening in contrast to mitochondria issued from other organs such as liver^36^. The greater resistance of cardiac mitochondria to Ca^2+^ overload might be linked to the contractile function of the heart which constantly involves large Ca^2+^ fluxes. It is also known that a moderate increase in intramitochondrial Ca^2+^ concentration influences energy production by stimulating several Kreb’s cycle enzymes. Therefore, cardiac mitochondria should be able to handle larger amount of Ca^2+^ to supply sufficient energy for contractile adaptation. This hypothesis is in accordance with previous data showing that interfibrillar mitochondria, which produce ATP for contractile function, are more resistant to Ca^2+^ overload than subsarcolemmal mitochondria^37^.

Contrasting with electroneutral ionophores, ETH129 induced a delayed and concentration-dependent hypercontracture of cardiomyocytes. This was associated with a loss of mitochondrial membrane potential preceding calcein loss by a few minutes. After calcein loss, cells quickly take up propidium iodide dye evidencing cell death. This might rely on intracellular Ca^2+^ stores as sarcoplasmic reticulum depletion with ryanodine greatly delayed calcein fluorescence decrease. These results support the existence of a rapid and permanent mPTP opening and are consistent with the ability of ETH129 to elicit mPTP opening in isolated mitochondria. Nevertheless, neither CsA treatment nor CypD deletion limited the decreases in calcein and TMRM fluorescence induced by ETH129 in cardiomyocytes. This shows that the effect of ETH129 is independent from a CypD-dependent mPTP opening process. However, it is well-established that CypD only facilitates mPTP opening. mPTP can form and open in the absence of CypD or in the presence of CsA when the inducing stimulus is sufficient^38^. This is probably the case with ETH129 which provokes a fast and high mitochondrial Ca^2+^ overload and concomitantly uncouples mitochondrial membrane potential. Indeed membrane depolarization was shown to facilitate mPTP opening although the mechanisms involved remain uncertain^39, 40^.

This study also reveals that hypoxia reoxygenation is an excellent model to highlight mPTP opening in cardiomyocytes. Matrix Ca^2+^ overload was the first factor described to activate mPTP opening^7, 41^ and is still considered as essential. However, other cellular regulatory factors such as oxidative stress, adenine nucleotide depletion or elevated phosphate concentrations are involved and proceed by enhancing the sensitivity of the mPTP to Ca^2+^ ^3, 4^. This is why a cellular model that takes into account these regulatory factors seems more relevant to study mPTP opening. Indeed, the reoxygenation of hypoxic cardiomyocytes is clearly associated with a CypD-dependent mPTP opening. This protocol has the advantage to combine Ca^2+^ overload with other factors such as oxidative stress which are known to enhance the mPTP sensitivity to Ca^2+^. Moreover, the cellular conditions generated by the hypoxic period (cellular Ca^2+^ overload, decrease in adenine nucleotide concentrations, high phosphate concentrations) facilitate mPTP opening during the reoxygenation phase.

In conclusion, Ca^2+^ ionophores are useful tools to induce mPTP opening in some cells. However, they cannot be used to trigger a CypD-dependent mPTP opening in cardiomyocytes either because the mechanism of Ca^2+^ transport at the level of the mitochondrial membrane is not effective enough (ionomycin, A23187) or the agent is too powerful (ETH129) and leads to a non-CypD regulated mPTP opening corresponding to what we observed during severe hypoxia-reoxygenation. In addition, the results of the present study demonstrate that the hypoxia-reoxygenation model is a suitable setting to highlight and investigate mPTP opening in cardiomyocytes.

## Material and Methods

### Reagents

All compounds were purchased from Sigma-Aldrich (Saint-Quentin Fallavier, France) unless specified.

### Animals

Male C57BL/6J mice (8 to 10 week-old) and male Wistar rats (250–300 g) were purchased from Janvier (Le Genest-St-Isle, France). CypD knock-out mice (*Ppif* ^−/−^ mice) were obtained from Jackson Laboratories (Bar Harbor, Maine, USA). Animals were housed in an air-conditioned room with a 12 h light–dark cycle and received standard rodent chow and drinking water *ad libitum*. All animal procedures used in this study were in accordance with the Directives of the European Parliament (2010/63/EU-848 EEC) and were approved by the Animal Ethics Committee Afssa/ENVA/Universite Paris Est Creteil (approval number 09/12/14-02).

### Isolation of cardiac mitochondria

Male Wistar rats were used for the preparation of cardiac mitochondria as previously described^25^. Briefly, hearts were homogenised for 5 s in a cold buffer solution (220 mM mannitol, 70 mM sucrose, 10 mM HEPES, 2 mM EGTA, pH 7.4 at 4 °C) using a Polytron homogeniser. Samples were then further homogenised for five consecutive times in a Potter-Elvehjem glass homogeniser at 1500 rpm. The homogenate was centrifuged at 1000 *g* for 5 min at 4 °C. The supernatant was centrifuged at 10000 *g* for 10 min at 4 °C. The mitochondrial pellet was resuspended in homogenization buffer without EGTA and protein concentration was determined using the Advanced protein assay reagent (Sigma catalog number #57697).

### Mitochondrial swelling assays

Mitochondrial swelling was assessed by measuring the change in absorbance at 540 nm (A_540_) using a Jasco V-530 spectrophotometer equipped with magnetic stirring and thermostatic control. In a first set of experiments, mitochondrial swelling induced by A23187 and ionomycin was measured in deenergised mitochondria. Experiments were carried out at 30 °C in a swelling buffer (100 mM KCl, 50 mM sucrose, 10 mM HEPES, 5 mM KH_2_PO_4_, pH 7.4 at 30 °C). Mitochondria (0.5 mg/mL) were incubated in the presence of rotenone, antimycine (1 µM each) and 200 µM Ca^2+^. After 30 s, swelling was induced by addition of 1 µM A23187 or 10 µM ionomycin.

In a second set of experiments, the effect of ETH129 on mitochondrial swelling was measured in energised conditions. Mitochondria (0.5 mg/mL) were incubated at 30 °C in a respiration buffer containing 100 mM KCl, 50 mM sucrose, 10 mM HEPES, 5 mM KH_2_PO_4_ (pH 7.4 at 30 °C) in the presence of pyruvate/malate (5/5 mM), ruthenium red (10 µM) and 200 µM Ca^2+^. After 30 s, swelling was induced by the addition of 10 µM ETH129. When used, cyclosporin A (CsA) was introduced at the beginning of the incubation period.

### Mitochondrial membrane potential assays

Mitochondrial membrane potential was evaluated by the uptake of the fluorescent dye rhodamine 123 which accumulates electrophoretically into energised mitochondria in response to their negative inner-membrane potential. Rhodamine 123 (0.2 µM) and the respiratory substrates pyruvate/malate (5/5 mM) were added to respiration buffer contained in a cuvette maintained at 30 °C. Fluorescence was monitored over time using a Jasco FP 6300 spectrofluorometer (excitation wavelength 503 nm; emission wavelength 527 nm). After 20 s of recording, mitochondria (0.4 mg/mL) were added in the cuvette and the effect of increasing concentrations of ETH129 on the membrane potential was examined after addition of the compounds at 120 s. Carbonyl cyanide p-trifluoromethoxyphenylhydrazone (FCCP, 1 µM) was added at the end of the experiment to depolarise mitochondrial membrane potential.

### Isolation of primary adult rat and mouse cardiomyocytes

Ventricular cardiomyocytes were isolated from mice and rats by an enzymatic technique. The heart was retrogradely perfused for 15 min at 37 °C with a stock perfusion buffer bubbled with 95%O_2_/5%CO_2_ containing 133 mM NaCl, 4.7 mM KCl, 0.6 mM KH_2_PO_4_, 0.6 mM Na_2_HPO_4_, 1.2 mM MgSO_4_, 12 mM NaHCO_3_, 10 mM KHCO_3_, 10 mM HEPES, 30 mM taurine, 0.032 mM phenol red, 5.5 mM glucose, 10 mM 2,3butanedionemonoxime pH 7.4 to wash out blood. After 2 min of perfusion, liberase (Blendzyme 10 mg/100 ml, Roche Applied Science, Mannheim, Germany), trypsin EDTA (14 mg/100 ml) and 12.5 μM Ca^2+^ were added to the buffer and the heart was perfused for approximately 8–9 and 13–15 min for mice and rats, respectively. The heart was placed into a beaker in the same buffer containing 10% bovine serum albumin pH 7.4 at 37 °C to stop the digestion. Ventricles were then cut into small fragments and cells isolated by stirring the tissue and successive aspirations of the fragments through a 10 ml pipette. After 10 min the supernatant was removed and the remaining tissue fragments were re-exposed to 10 ml of the same buffer. Then, the cells were suspended in the same buffer and Ca^2+^ was gradually added to 1 mM into an incubator at 37 °C. Finally, the cardiomyocytes were suspended in culture medium M199. They were seeded on 35 mm Petri dishes pre-coated with 10 μg/ml sterilised laminin and incubated for 90 min before being used.

### Measurement of mPTP opening, membrane potential, cell death and mitochondrial Ca^2+^ in cells

**Figure 7:**
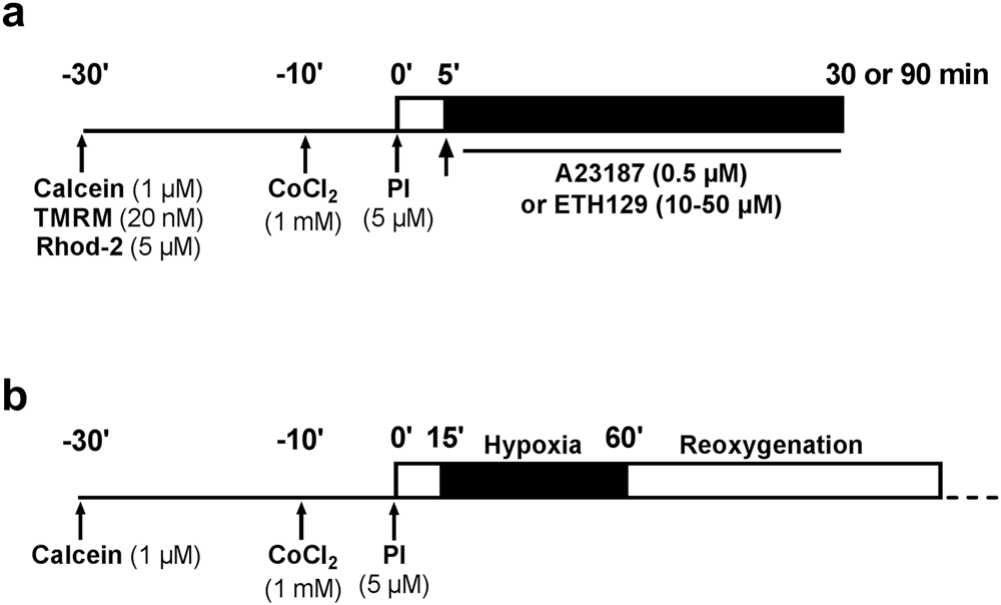
Design of the experiments performed on isolated cardiomyocytes.

Assessment of mPTP opening in cardiomyocytes was performed using the established calcein cobalt loading procedure by incubating cells with calcein-acetoxymethyl ester (calcein-AM) and CoCl_2_ which results in calcein fluorescence localised in mitochondria^15, 24^. Cells were loaded with 1 μM calcein-AM for 30 min at 37 °C in 2 ml of M199, pH 7.4, supplemented with 1 mM CoCl_2_ and then washed free of calcein and CoCl_2_ and M199 was replaced by a Tyrode’s buffer (in mM: NaCl 130; KCl 5; HEPES 10; MgCl_2_ 1; CaCl_2_ 1.8, pH 7.4 at 37 °C). When the mitochondrial membrane potential was analysed, cardiomyocytes were loaded with 20 nM tetramethylrhodamine methyl ester (TMRM) for 30 min at 37 °C and then rinsed with the Tyrode’s solution. To monitor mitochondrial Ca^2+^, cardiomyocytes were loaded with 5 µM rhod-2 AM (Thermofisher Scientific, Villebon-sur-Yvette, France) for 40 min at 37 °C and then washed free of rhod-2 AM. When cell death was studied, propidium iodide (5 μM) which permeates only the damaged cells was introduced in the Tyrode’s buffer. To induce mPTP opening, calcium ionophores were added to the medium after 5 min stabilization, and remained present until the end of the measure (Fig. 7a). Cardiomyocytes were imaged with an Olympus IX-81 motorised inverted microscope equipped with a mercury lamp as a source of light for epifluorescence illumination and with a cooled camera (Hamamatsu ORCA-ER). For the detection of calcein fluorescence, a 460–490 nm excitation and a 510 nm emission filter were used. Propidium iodide and TMRM fluorescence were excited at 520–550 nm and recorded at 580 nm. For the detection of rhod-2 a 552 excitation and a 581 nm emission filter were used.

In normoxic experiments, images were acquired every 1 or 5 min. In hypoxia-reoxygenation experiments, images were acquired every 5 min during hypoxia, every min during the first 5 min following reoxygenation and then every 5 min. The illumination times were of 25 ms (calcein), 70 ms (propidium iodide), 50 ms (TMRM) and 70 ms (rhod-2). Images were analyzed using a digital epifluorescence imaging software (XCellence, Olympus, Rungis, France). Fluorescence was integrated over a region of interest (≈80 μm^2^) for each cardiomyocyte and a fluorescence background corresponding to an area without cells was subtracted. For comparative purposes, the fluorescence intensity minus background was normalised according to the maximal fluorescence value (initial value for calcein, TMRM and rhod-2, final value for propidium iodide).

For each experiment, the change of fluorescence was observed from a single cardiomyocyte and then, the global response was analysed by averaging the fluorescence changes obtained from all the cardiomyocytes (at least 30 cells) contained in a single field. In hypoxia-reoxygenation experiments, the time of reoxygenation necessary to induce a 50% decrease in calcein fluorescence (time to 50% mPTP opening [T_mPTP50_]) and to induce a 50% increase in propidium iodide fluorescence (time to 50% cell death [T_cell death50_]) were determined. When cell contracture was evaluated, cells were considered hypercontracted when they exhibited an irreversible loss of the rod-like shape (Fig. 6a). Hypercontracture was expressed as the percentage of contractured cells over the total cells on the field.

### Hypoxia-reoxygenation model

Mouse cardiomyocytes were placed into a thermostated (37 °C) chamber (Warner Instruments Inc, Connecticut) which was mounted on the stage of an IX81 Olympus microscope (Olympus, Rungis, France) and were perfused with the Tyrode’s solution at a rate of 0.5 ml/min. The chamber was connected to a gas bottle diffusing a constant stream of O_2_ (21%), N_2_ (74%) and CO_2_ (5%) maintaining an O_2_ concentration of 21%. Oxygen in the perfusate was measured in the chamber using a fibre optic sensor system (Ocean Optics Inc., Florida). Cardiomyocytes were paced to beat by field stimulation (5 ms, 0.5 Hz).

To simulate ischaemia, the perfusion was stopped and cardiomyocytes exposed for 45 or 90 min to a hypoxic medium maintaining an O_2_ concentration of 1–2%. This medium was the Tyrode’s solution (bubbled with 100% N_2_) supplemented with 20 mM 2-deoxyglucose and subjected to a constant stream of N_2_ (100%). At the end of the ischemic period, reoxygenation was induced by rapidly restoring the Tyrode’s flow and 21% O_2_ in the chamber (Fig. 7b).

### Statistical analysis

The data are reported as mean ± S.E.M. Statistical significance was determined using either Student’s two-tailed unpaired *t*-test or one-way analysis of variance (ANOVA) followed by Newman-Keuls’ post test. Significance was accepted when p < 0.05.
